# Piloting of a modular competency-based training to improve maternal and newborn health in Nepal: a mixed-methods study

**DOI:** 10.1093/inthealth/ihag039

**Published:** 2026-04-23

**Authors:** Yeshoda Aryal, Khim Bahadur Khadka, Deepak Paudel, Chetan Nidhi Wagle, Ishwar Upadhyaya, Rajendra Bhadra, Surya Bhatta, Olena Ivanova, Guenter Froeschl

**Affiliations:** National Health Training Centre (NHTC), Ministry of Health and Population, Teku, Kathmandu, Nepal; Centre for International Health (CIH), LMU Munich, Leopoldstraße 13, 80802 Munich, Germany; Provincial Health Directorate, Ministry of Health, Gandaki Province, Pokhara, Nepal; Centre for International Health (CIH), LMU Munich, Leopoldstraße 13, 80802 Munich, Germany; National Health Training Centre (NHTC), Ministry of Health and Population, Teku, Kathmandu, Nepal; National Health Training Centre (NHTC), Ministry of Health and Population, Teku, Kathmandu, Nepal; Independent Expert, Kathmandu, Nepal; One Heart Worldwide, Kathmandu, Nepal; Centre for International Health (CIH), LMU Munich, Leopoldstraße 13, 80802 Munich, Germany; Institute of Infectious Diseases and Tropical Medicine, University Hospital, Ludwig Maximilian University, Munich, Germany; SPHERE, National Health and Medical Research Council Centre of Research Excellence, Melbourne, VIC, Australia; Department of General Practice, Monash University, Building 1, 270 Ferntree Gully Road, Notting Hill, VIC 3168, Australia; Centre for International Health (CIH), LMU Munich, Leopoldstraße 13, 80802 Munich, Germany; Institute of Infectious Diseases and Tropical Medicine, University Hospital, Ludwig Maximilian University, Munich, Germany

**Keywords:** competency-based education, health personnel, maternal health services, Nepal, newborn, quality of healthcare

## Abstract

**Background:**

Despite increased facility-based childbirth in Nepal, stagnant neonatal and high maternal mortality highlight a critical ‘know–do’ gap in care quality. To address this, a modular, competency-based learning resource package (LRP) was developed for maternal and newborn health providers.

**Methods:**

A mixed-methods exploratory pilot study involved curriculum alignment with international standards, multidisciplinary co-design and a single-group pre–post evaluation among 24 nursing professionals in Gandaki Province. Feasibility and acceptability were evaluated utilizing objective structured clinical examinations (OSCEs) and qualitative interviews.

**Results:**

Non-parametric analyses revealed significant educational gains. Median knowledge scores increased from 64.0 to 88.0. Statistically significant median improvements (p<0.001) were documented across all OSCE clinical domains: antenatal care (30.0 to 92.1), postnatal care (53.8 to 90.4), infection prevention (31.8 to 90.0) and respectful maternity care (30.0 to 92.9). Qualitative data indicated high acceptability; however, heavy clinical workloads and linguistic diversity constrained programmatic sustainability.

**Conclusions:**

The LRP is a feasible intervention to bridge clinical competency gaps. Definitive national scale-up requires larger controlled trials assessing patient-level outcomes along with essential systemic investments in supply chains, healthcare infrastructure and continuous post-training mentorship.

**List of Abbreviations**
ANM: Auxiliary Nurse Midwife
CI: Confidence Interval
COREQ: Consolidated Criteria for Reporting Qualitative Research
ICM: International Confederation of Midwives
IQR: Interquartile Range
LMICs: Low- and Middle-Income Countries
LRP: Learning Resource Package
MNH: Maternal and Newborn Health
NHTC: National Health Training Centre
OSCE: Objective Structured Clinical Examinations
RMNC: Respectful Maternity and Newborn Care
SBA: Skilled Birth Attendant
SHP: Skilled Health Personnel
STROBE: Strengthening the Reporting of Observational Studies in Epidemiology
TWG: Technical Working Group
WHO: World Health Organization

## Introduction

The quality of intrapartum care is a critical determinant of maternal and newborn health.^1,2^ Across many low- and middle-income countries (LMICs), a rapid increase in facility-based childbirth has not yielded a corresponding decline in mortality. This reflects an access–quality gap often termed the ‘stagnation paradox’, whereby increasing facility deliveries have not translated into proportional improvements in outcomes.^1,3^

Nepal reflects this pattern. Over the past 2 decades, the country has achieved a remarkable increase in institutional delivery coverage (from 8% in 1996 to 79% in 2022), driven by strategic initiatives such as establishing birthing centres, implementing maternity incentive schemes and deploying skilled birth attendants (SBAs).^4,5^ During the same period, substantial reductions in maternal mortality (from 539 to 151 per 100 000 live births between 1996 and 2021) and newborn mortality (from 50 to 21 per 1000 live births) were noted; however, as coverage increased, nearly 57% of maternal deaths now occur within facilities.^4,6^ This underscores that access alone does not ensure survival; provider competence and quality of care must improve concurrently to reduce preventable deaths.^1,3^

The effectiveness of SBAs in reducing maternal and newborn mortality depends on their clinical and interpersonal competencies, which are acquired through pre-service education, in-service training and ongoing mentorship and support for continued learning and clinical practice.^1,2^ However, evidence from Nepal and other LMICs indicates that many graduates of standard SBA training do not consistently meet the required competency standards, particularly in terms of clinical skills.^7^ This deficiency is often attributed to traditional, didactic teaching models that prioritize theoretical knowledge over the application of psychomotor skills. This gap between training and real-world competencies, often referred to as the ‘know–do’ gap, remains a persistent barrier to quality care.^7,8^

Addressing these training gaps requires aligning national capacity-building efforts with the national and international competency frameworks. While the World Health Organization (WHO) and the International Confederation of Midwives (ICM) have outlined clear quality standards, guidance on translating these into context-specific training models remains limited.^2,9^ To bridge this gap, experts have increasingly advocated for competency-based simulation-rich approaches complemented by post-training mentorship.^8,10^ Simulation-based curricula bridge the ‘know–do’ gap by focusing on Miller’s ‘Shows How’ level, ensuring that providers demonstrate the psychomotor mastery required for obstetric emergencies. When integrated into competency-based frameworks, this modular approach aids the transition from simulated proficiency to real-world clinical practice.^11^

Nepal’s maternal and newborn health (MNH) strategies,^12,13^ ICM competencies and WHO guidelines^9,14^ reflect a global shift toward competency-based education to improve provider competence in low-resource settings.^15,16^ Evidence shows that hands-on, competency-based training improves safety, quality and outcomes.^17–19^ Research also indicates that short, frequent practice sessions coupled with on-site mentoring assist teams in maintaining skills.^20^ However, evidence from Nepal suggests that training effectiveness diminishes when supervision and supplies are lacking.^21^ Furthermore, while tools such as the WHO Labour Care Guide are highly usable, their application remains uneven.^22^ Recognizing that generic SBA training has not yielded consistent competence, the National Health Training Centre (NHTC) has developed a modular, simulation-rich learning resource package (LRP) for skilled health personnel/skilled birth attendants (SHP/SBAs),^23^ explicitly aligned with WHO quality-of-care standards and ICM essential competencies,^9,14^ to provide a feasible, context-adapted model for enhancing the MNH workforce. This exploratory pilot study provides a concrete assessment of this novel cadre-tailored pedagogical framework designed to transcend the limitations of traditional didactic methods. Specifically, we conducted an exploratory pilot to operationalize international standards into a feasible, modular curriculum tailored to specific provider professional roles (auxiliary nurse midwives [ANMs] and staff nurses); pilot the LRP to assess the feasibility and acceptability of this cadre-specific approach; and estimate preliminary gains in psychomotor clinical performance and respectful care behaviours while documenting fidelity and key resource/logistical requirements for scale-up.

## Methods

### Study design

This study used a three-phase exploratory pilot design. Phase 1 focused on assessing standard SBA training to show gaps and training approaches. Phase 2 documented the participatory development of the modular SHP/SBA LRP. Phase 3 involved pilot testing the LRP with 24 maternal newborn care providers (ANMs and staff nurses) at two sites (Matrisishu Miteri and Lamjung Hospitals in Gandaki Province) to assess its feasibility, acceptability and preliminary effects on providers’ knowledge and skills. By updating the traditional one-size-fits-all course with simulation-enhanced, competency-based modular training tailored to different provider cadres, the LRP intends to target the ‘know–do’ gap and is expected to contribute to further gains in MNH outcomes.^8,23^ This study followed the conceptual framework outlined in Supplementary Figure S2.

#### Phase 1: Assessment of the standard SBA training

Phase 1 focused on assessing the structure, content, delivery and effectiveness of Nepal’s standard SBA in-service training, with particular attention to the implementation experiences in Gandaki Province. We systematically mapped the standard SBA training curriculum (learning aims, instructional hours, teaching methods and assessments) against the 2024 ICM Essential Competencies for Basic Midwifery Practice^9^ using a structured checklist to identify the gaps. We also conducted structured workshops and in-depth interviews with 12 stakeholders (SBAs, SBA trainers, program managers and policymakers). The sessions were audio recorded and transcribed. Two analysts independently coded the transcripts using inductive thematic analysis^24^ and any differences were resolved by consensus.

#### Phase 2: Development of the modular SHP/SBA LRP

Phase 2 focused on developing a modular LRP through a participatory, evidence-based process spearheaded by the NHTC in Nepal. The formulation of the LRP was directly informed by the deficiencies and requirements identified in phase 1, as well as an analysis of global and national evidence of best practices. The LRP was drafted through a consultative process involving a nine-member technical working group (TWG) comprising multidisciplinary experts, including obstetricians, midwifery educators, pedagogical specialists and researchers. Between February 2023 and May 2025, six TWG meetings were held to review and refine the successive drafts. The TWG feedback, particularly on clinical relevance, feasibility and pedagogy, was recorded and incorporated into subsequent drafts. The process continued until a consensus was reached on the LRP content and structure, concluding with TWG endorsement. Broader feedback was obtained through three national consultative workshops (April 2023, August 2023 and April 2024) with MNH experts, policymakers and senior trainers. This participatory validation process ensured that the LRP was contextually appropriate and aligned with national priorities. The TWG consensus yielded four key design decisions responding directly to phase 1 gaps: introducing flexible cadre-specific pathways, shifting to simulation-based psychomotor training, mandating a foundational respectful maternal and newborn care (RMNC) module and integrating structured post-training mentorship programs.

#### Phase 3: Pilot testing of the modular SHP/SBA LRP

Phase 3 involved the pilot implementation of the LRP to assess its feasibility, acceptability, fidelity and training site readiness (e.g. logistical needs, trainer availability), as well as preliminary outcome effectiveness. Pilot training was conducted at two sites in Gandaki Province from June to July 2025, after training trainers in May 2025. A purposive sample of 24 participants was recruited from the total institutional pool of MNH providers at the selected facilities. Inclusion criteria required participants to be currently active in clinical MNH service delivery (birthing centres or maternity wards) at the time of the study. Providers in purely administrative roles or those on long-term leave were excluded. The purposive selection^25^ was operationalized in coordination with district public health offices to ensure a representative mix of professional cadres, specifically staff nurses and ANMs, across varying levels of clinical experience and facility types. The sample size of 24 was determined based on the logistical capacity of high-fidelity simulation laboratories (as per training protocol limited to 12 participants per cohort) and was considered appropriate for an exploratory pilot study focused on assessing intervention feasibility and acceptability rather than formal statistical power for population-level representativeness. Eligible participants had no prior standard SBA training. All 24 invited providers agreed to take part, resulting in no refusals or dropouts. All participants provided informed written consent. The LRP modules were delivered over a period of 36 d (approximately 252 h), including module 1 (12 d; 84 h), module 2 (4 d; 28 h) and module 3 (20 d; 140 h). These modules covered foundational RMNC and core skills (module 1), emergency obstetric and newborn first aid (module 2) and the physiology and management of normal labour and birth (module 3). The curriculum was delivered through classroom sessions, simulation exercises and clinical skill practices, facilitated by expert trainers.

### Assessment measures and data analysis

Quantitative and qualitative data were collected for this study. The assessment used a single-group pre–post design, a method chosen to be pragmatic and appropriate for this study. The primary focus was to assess the feasibility, acceptability and preliminary outcomes of a new modular training course before committing to a larger, resource-intensive controlled trial. Furthermore, a concurrent control group was considered inappropriate for this pilot phase because of ethical considerations regarding withholding potentially beneficial training and significant logistical constraints of the study setting. Knowledge was assessed pre- and post-training, using parallel forms of a multiple-choice test that was content confirmed by the TWG to ensure alignment with the LRP modules. Skills were assessed using objective structured clinical examination (OSCE) stations developed de novo by the TWG, with scenarios and checklists explicitly mapped to the ICM international standards.

The OSCE development process involved iterative refinement through expert consensus and pilot testing to ensure content validity and reliability (Supplementary File S4). The performance was scored by expert trainers using standardized rubrics to ensure consistency (Supplementary File S5). However, certain bias-control measures typical of definitive trials, such as assessor blinding and the use of independent external raters, were not implemented in this pilot study. The assessors consisted of the LRP training faculty who were aware of the participants’ pre- and post-intervention status. Although formal interrater reliability coefficients were not statistically calculated, scoring consistency was promoted through the application of highly detailed, binary standardized rubrics and a consensus-based scoring approach among the trainers during daily debriefings. The proficiency pass mark for the theoretical knowledge test was set at 85%. For clinical skills assessed via OSCEs, the mastery threshold was strictly set at 100%, reflecting the critical, life-saving nature of the intrapartum and neonatal interventions where omitted steps compromise patient safety.

Data normality was assessed using the Shapiro–Wilk test. As the score distributions for knowledge and all clinical skill components significantly violated normality assumptions (p<0.05) primarily due to an expected educational ceiling effect after the intervention, non-parametric Wilcoxon signed-rank tests were used for pre–post comparisons using Stata/SE 19 (StataCorp, College Station, TX, USA). The aggregate change in the composite clinical competency (OSCE) score was chosen as the primary endpoint for evaluating the preliminary effectiveness of the modular LRP. Descriptive statistics are presented as medians and interquartile ranges (IQRs). To ensure the precision of the intervention’s effect, exact 95% confidence intervals (CIs) were calculated for all median differences. Consistent with the exploratory pilot design and limited sample size (N=24), the statistical analysis was restricted to aggregate cohort-level changes. Adjustments for covariates, subgroup comparisons and sensitivity analyses were intentionally omitted to maintain inferential clarity and avoid overinterpretation of underpowered associations.

The semi-structured interview guides were developed iteratively by the core research team, informed by the study objectives and gap analysis findings from phase 1. Qualitative data were analysed using thematic analysis following the six-phase recursive approach proposed by Braun and Clarke,^24^ which was selected for its flexibility in deriving actionable insights within exploratory health services research. The coding steps were performed manually using Excel (Microsoft, Redmond, WA, USA). The coding steps included familiarization with the data, generating initial codes, searching for themes, reviewing themes against the dataset, defining and naming themes and producing the final report. Purposeful recruitment ensured representation across the professional cadres. Thematic saturation was established when successive interviews yielded no new codes. To ensure reflexivity and minimize bias, the coding process incorporated regular team debriefings and intercoder consensus discussions. The reporting of the quantitative part conformed to the Strengthening the Reporting of Observational Studies in Epidemiology guidelines. Qualitative reporting adhered to the Consolidated Criteria for Reporting Qualitative Research checklist (Supplementary File S3).

### Declaration of artificial intelligence (AI) use

Following journal editorial policies, AI tools (Elicit, Paperpal, Gemini) were used strictly for literature retrieval assistance, structural editing and data formatting. No original scientific content or statistical analysis was generated by AI.

## Results

### Phase 1: Assessment of the standard SBA training

#### Strengths of standard SBA training

Standard training uses competency-based materials aligned with national policies and international (ICM and WHO) guidelines, supported by a quality improvement system via the NHTC. The training duration is adapted based on participants’ baseline qualifications, and a follow-up enhancement program reflects a commitment to quality assurance.

#### Competency gaps in the training curriculum in relation to ICM essential competencies

Despite these strengths, significant competency gaps were clear among standard SBA trainees. Mapping the curriculum revealed that, on average, only 45% of the content met the 2024 ICM benchmarks for basic midwifery, with notable underperformance in decision-making and practical skill execution. Furthermore, health management information system data showed that many trained providers do not meet the WHO-recommended minimum supervised childbirths, showing insufficient clinical exposure to supervised childbirths.

Stakeholder consultations and curriculum analyses highlighted several systemic areas requiring improvement, which are summarized in Table 1. A primary concern was the lack of cadre differentiation resulting in an overloaded, didactic curriculum. As one SBA trainer noted during the consultations:

**Table 1:** Summary of identified gaps in standard SBA training and implications for LRP design.

Key gaps identified	Examples from assessment	Implications for modular LRP design
Lack of cadre differentiation	A standardized training program is implemented indiscriminately for all front-line providers, resulting in an overloaded curriculum (27 core skills) in a compressed time	Develop flexible, cadre-specific learning pathways tailored to different provider categories (e.g. ANMs vs staff nurses)
Insufficient practical skill mastery	Trainees have restricted hands-on practice due to low patient volume and didactic teaching; many do not meet WHO-recommended supervised childbirth minimums	Shift to a simulation-based, hands-on method to focus on psychomotor skills and emergency management
Inadequate focus on respectful care	The curriculum inadequately addresses the care continuum, specifically RMNC and communication skills	Incorporate a mandatory foundational module dedicated to RMNC, communication and woman-centred practice
Weak post-training support	Lack of ongoing mentoring and coaching and poor supervision leads to rapid skill decline over time	Integrate structured post-training mentorship and coaching pathways to mitigate skill attrition

We are delivering the exact same heavy curriculum to everyone, which leaves very little time for actual hands-on practice. By the end of the course, many still lack the confidence to manage real emergencies. (Stakeholder 04, SBA trainer)

Furthermore, stakeholders emphasized that restricted hands-on practice was compounded by weak post-training support. Another participant highlighted this barrier:

Once the training is over, there is virtually no mentorship at the facility level. Without ongoing coaching and supervision, their clinical skills quickly decline. (Stakeholder 09, program manager)

The detailed gaps and their corresponding implications for the newly designed LRP are outlined below.

### Phase 2: Key features of the modular SHP/SBA LRP

A new modular LRP was designed to address the gaps found in phase 1 of this study. It provides flexible, context-specific and cadre-specific training aimed at strengthening MNH services. Key features included a strong emphasis on respectful, woman-centred care, enhancement of essential midwifery competencies and the WHO Labour Care Guide. The LRP aimed to improve providers’ ability to manage normal labour, promptly find and manage complications and manage obstetric and newborn emergencies in the future. It also incorporates specific adaptations for workers in low-resource and remote settings. The LRP focused on simulation-based practice and tailored learning pathways for different provider categories and reinforced respectful care practices. The five modules covered were as follows: foundational, including continuum of care (RMNC); emergency management (first aid/stabilization for obstetric/newborn emergencies); normal labour and delivery care; management of complications (e.g. postpartum haemorrhage, pre-eclampsia) and an advanced module for doctors (focusing on operative delivery and advanced newborn care). Each module incorporated simulation and case-based learning, with mastery evaluation using OSCE stations and checklists. Mentorship and coaching pathways were integrated to mitigate post-training skill attrition (Supplementary Table S1).

### Phase 3: Piloting of the LRP

#### Participant characteristics

All 24 participants completed the 36-day pilot training (100% completion rate). The cohort was drawn from seven districts in Gandaki Province, being a diverse range of ethnic backgrounds and facilities. The participants included 15 ANMs and 9 staff nurses, with the majority employed at health posts and basic health centres. This demographic and professional heterogeneity shows that the pilot sample adequately represented the various tiers of Nepal’s primary MNH care system. Although specific data on the participants’ exact years of clinical experience were not collected, all selected individuals were actively working in the maternity field and had no prior standard SBA training. Table 2 provides a detailed summary of participant characteristics.

**Table 2:** Background characteristics of the participants (n=24).

Characteristics	Category	n (%)
Ethnicity	Janjati	11 (45.8)
	Brahmin/Chhetri	9 (37.5)
	Madhesi	2 (8.3)
	Dalit	2 (8.3)
Qualification	ANM (including senior ANM)	15 (62.5)
	Proficiency certificate–level nursing (staff nurse)	9 (37.5)
Designation	ANM	19 (79.1)
	Staff nurse	5 (20.8)
District	Syangja	5 (20.8)
	Parbat	4 (16.7)
	Lamjung	4 (16.7)
	Gorkha	3 (12.5)
	Tanahun	3 (12.5)
	Myagdi	3 (12.5)
	Baglung	2 (8.3)
Health facility type	Health post/primary health centre	10 (41.7)
	Basic health centre (including birthing centre, urban health clinic)	9 (37.5)
	Community health unit	3 (12.5)
	Primary hospital	2 (8.3)

#### Knowledge outcomes

The participants showed a significant increase in theoretical knowledge following the intervention. The median knowledge scores increased from 64.0 (IQR 60.0–72.0) to 88.0 (IQR 88.0–92.0), being a median improvement of 24.0 percentage points (95% CI 20.0 to 28.0, p<0.001), indicating immediate knowledge gains attributable to the LRP.

#### Clinical skills outcomes

Table 3 summarizes the changes in the participants’ clinical skills following the LRP intervention. Statistically significant improvements were seen in all four assessed domains (p<0.001). When evaluated against predefined absolute mastery standards, 100% (24/24) of participants achieved the 85% pass mark for theoretical knowledge post-intervention. For clinical competency evaluated against a strict 100% mastery threshold, 100% (24/24) of participants successfully completed all critical steps for antenatal care, 58.3% (14/24) for postnatal care, 70.8% (17/24) for infection prevention and 95.8% (23/24) for respectful maternity care, demonstrating strict adherence to life-saving clinical protocols.

**Table 3:** Pre- and post-training competency scores (n=2).

Competency domain	Pre-test score, median (IQR)	Post-test score, median (IQR)	Difference, median (95% CI)	p-Value
Knowledge	64.0 (60.0–72.0)	88.0 (88.0–92.0)	24.0 (20.0 to 28.0)	<.001
Antenatal care	30.0 (20.0–60.0)	92.1 (89.5–97.4)	63.4 (48.5 to 69.7)	<.001
Postnatal care	53.8 (46.2–61.5)	90.4 (78.8–93.3)	30.1 (27.8 to 37.6)	<.001
Infection prevention	31.8 (20.5–45.5)	90.0 (80.0–100.0)	53.2 (44.5 to 57.3)	<.001
Respectful care	30.0 (12.5–37.5)	92.9 (89.3–95.5)	64.3 (57.1 to 79.5)	<.001

The cumulative emergency OSCE (module 2) showed the participants’ proficiency in time-critical obstetric and neonatal interventions. All participants completed the assessments within the designated period. Station-specific analysis revealed universal competency (100% [24/24]) in both resuscitation and essential newborn care. High proficiency was similarly observed for postpartum haemorrhage management (95.8% [23/24]) and management of severe pre-eclampsia/eclampsia (91.7% [22/24]). Furthermore, proficiency in the active management of the third stage of labour improved markedly from 35% at baseline to 95% post-intervention.

### Qualitative findings

#### Feasibility and acceptability of the intervention

The course completion rate was 100% (n=24) with full adherence to the training schedule. The participants universally confirmed the relevance of the curriculum and acceptability of the modular approach. Although training venues were well equipped, resource constraints on newborn mannequins were mitigated through the implementation of rotational small-group stations. Participants (n=18) supported the simulation-rich approach, noting its practical utility. One participant highlighted this pedagogical shift:

The simulation helped me transfer my knowledge from my head to my hands, making me feel ready for real cases. (P04, staff nurse)

However, providing a cautious perspective on sustainability, several providers (n=5) noted the systemic barriers to implementation. Furthermore, language barriers were identified as a constraint, with one participant noting,

The materials are great, but having the technical terms entirely in English makes it harder for some of the older ANMs to grasp quickly. (P12, ANM)

The mixed-cadre composition (ANMs and staff nurses) successfully fostered teamwork. However, observational data have shown that senior staff members often assume leadership roles in complex scenarios.

#### Relevance to practice and self-efficacy

Participants (n=4) reported that the training effectively bridged the gap between theoretical knowledge and its clinical application:

This training taught me to care for the mother as a whole person, not just focus on the delivery process. The communication skills module was particularly valuable. (P12, ANM)

Simulation exercises were identified as the primary mechanism for this shift, enabling learners to move from theoretical understanding to practical application of knowledge.

#### Impact on respectful maternity and newborn care

The RMNC module was highly regarded for reinforcing communication skills and woman-centred practice. Participants (n=6) described a paradigm shift in their perspective, emphasising the necessity of holistic care. As one participant noted, the curriculum instilled a ‘mandate to care for the mother, not just deliver the baby’ (P10, ANM), thereby reinforcing a professional commitment to compassionate and dignified care.

#### Barriers to implementation and sustainability

Participants (n=5) identified specific pedagogical and systemic barriers to long-term skill retention and application in the workplace. Regarding implementation barriers, one participant noted:

The English materials were sometimes difficult to follow quickly during practice. Having Nepali versions would help us learn faster and feel more confident. (P15, staff nurse)

Additionally, participants (n=7) requested increased time allocation for practising high-complexity skills such as newborn resuscitation. Beyond the training environment, heavy clinical workloads and supply chain deficiencies at participants’ (n=8) home facilities were cited as major impediments to implementing new skills. However, challenges to sustainability were noted:

While the training was comprehensive, lack of consistent supplies at my facility sometimes makes it difficult to apply every standard step in an emergency. (P08, ANM)

## Discussion

This exploratory pilot study suggests that a modular, simulation-rich training program can narrow the gap between theoretical knowledge and clinical competence in MNH care, suggesting that it is a feasible and promising approach for diverse cadres and facilities in Nepal. While this suggests a potential reduction in the ‘know–do’ gap, participants indicated that supportive systems, including mentorship, adequate infrastructure and language-appropriate materials, are essential for sustaining these skills and ensuring an equitable impact.

First, the LRP’s interactive competency-based design helped translate theoretical knowledge into practical skills. The significant post-training gains in both knowledge and skills align with global evidence that simulation-based, hands-on modules bridge the gap between classroom learning and clinical practice.^26,27^ This was exemplified by one participant’s reflection that the simulation practice shifted their confidence ‘from my head to my hands’, highlighting the value of the hands-on methodology. Our findings support the hypothesis that well-designed practice-oriented training can close the ‘know–do’ gap in LMIC settings.

Second, the participants reported that the LRP was feasible and acceptable. All 24 invited providers completed the training and provided positive feedback on the training’s relevance. Participants valued the focus on communication, with one noting that it taught them ‘to care for the mother, not just deliver the baby’, underscoring the integration of critical interpersonal skills. This mirrors the findings from other simulation-based MNH training programs in LMICs, which are often well received and improve not only knowledge but also teamwork and communication.^28^ The positive reception across different cadres and facility types suggests that the curriculum is well-suited to Nepal’s public healthcare delivery system. However, any national scale-up must be contingent upon the results of future, larger-scale, controlled implementation trials.

Third, the sustainability and equity of the training’s impact depend on supportive systems. Our qualitative findings highlighted persistent barriers, such as heavy workloads, shortages of simulation equipment and English-only training materials. These challenges threaten long-term skill retention and corroborate phase 1 findings, in which stakeholders found the absence of post-training mentorship as the primary driver of skill decay. Multicountry reviews have found that knowledge and skills decline within 1 y unless refreshed.^29^ Embedding follow-up mentorship and refresher sessions is critical, as evidence shows that it reinforces learning and improves provider competency.^30^ Similarly, a lack of basic supplies and simulation models are major barriers to quality care.^26^ Finally, adapting content to the local language and context is more effective.^31^

To address these challenges, our findings highlight the need for a context-adapted mentorship model for Nepal that moves beyond generic post-training follow-up. Such a model should be built on three core principles derived from our qualitative data: language-appropriate support, with all mentorship tools and refresher materials provided in Nepali to maximise comprehension and use (ensuring ‘linguistic equity’); workflow-integrated coaching, using low-dose, high-frequency practice sessions that accommodate heavy clinical workloads and reinforce skills in the real-world environment; and resource-adaptive problem solving, where mentors help providers apply competencies in settings with inconsistent supplies and equipment. This structured approach offers a more robust strategy for sustaining LRP gains and ensuring long-term impact. Crucially, educational interventions cannot occur in a vacuum; scale-up must be strictly coupled with supply chain strengthening and local infrastructure investments (e.g. ensuring oxytocin and resuscitator availability) to prevent rapid skill decay in the clinical environment.

### Limitations and strengths of the study

As an exploratory pilot, this study had several limitations inherent to an uncontrolled, single-group, pre–post design. The sample was small (n=24), non-random and from a single province, which limited the external validity and causal inference. Additionally, potential observer bias and test–retest familiarity were acknowledged as trainers conducted the OSCE assessments, although this was mitigated using strict, standardized scoring rubrics. The outcomes were measured immediately after training, thus the durability was unknown. The absence of a control group means that the Hawthorne effect could not be ruled out. Specifically, the significant gains seen in interpersonal competencies, such as the RMNC, must be interpreted with caution. Participants’ awareness of being evaluated during the OSCEs may have temporarily induced positive behavioural compliance rather than permanent practice modification. Consequently, longitudinal, unobserved follow-up is necessary to confirm whether these results reflect lasting behavioural changes.

Furthermore, this pilot study exclusively assessed provider competency, and direct clinical and patient-level outcomes (e.g. maternal and neonatal mortality) were not measured. Numerous pre–post evaluations carry an inherent risk of type I error inflation due to multiple comparisons. A larger, multiprovince, cluster-randomised trial or alternative designs such as stepped-wedge or wait-list control trials, with a longer follow-up period and economic evaluation, are needed to confirm the effects on competencies, clinical practice and MNH outcomes. Furthermore, the universal proficiency observed post-intervention suggests a potential ceiling effect, highlighting the need for more discriminating or higher-complexity OSCE stations in future trials to better capture the variance in skill acquisition.

Despite these limitations, this exploratory pilot study employed a systematic, multiphase design and mixed-methods approach, integrating diverse stakeholder perspectives and supplementing qualitative insights with quantitative pre- and post-assessments. The participatory LRP development process engaged national experts and end users, improving contextual fitness and alignment with local and national priorities and increasing the plausibility of scaling up. The study followed an iterative process; e.g. qualitative findings from phase 1 found training delivery gaps and phase 3 showed gains in provider knowledge and skills immediately after training, offering valuable triangulated insights.

### Implications for policy, practice and research

While these exploratory findings are highly promising, any national scale-up of the competency-based LRP must be strictly contingent upon the results of future larger-scale controlled implementation trials that evaluate long-term skill retention and direct patient-level outcomes. Key priorities for future implementation include developing a train-the-trainer system, accrediting training sites and ensuring adequate simulation equipment and post-training mentorship. Furthermore, educational interventions cannot operate in isolation; to prevent rapid skill decay in the clinical environment, training scale-up must be strictly coupled with supply chain strengthening and local facility infrastructure investments (e.g. ensuring the uninterrupted availability of essential commodities such as oxytocin and neonatal resuscitators). Sustainable financing is vital for translating provider skills into better population health outcomes. We hypothesize that this competency-based, simulation-focused and mentorship-supported approach could serve as a transferable framework for other LMICs, provided it is contextually adapted and aligned with WHO and ICM quality care standards.

## Conclusions

This exploratory pilot study suggests that the modular, competency-based LRP is a feasible and promising approach to improving provider competency. Future large-scale controlled trials are needed to show a definitive link between training and clinical outcomes. Consequently, any definitive national scale-up must remain contingent upon the empirical validation of these future trials and must be inherently coupled with investments in facility infrastructure, supply chain strengthening and continuous post-training mentorship programs. We hypothesize that Nepal’s experience offers a promising framework for context-specific adaptation in other LMICs. Future research should evaluate the long-term effects of this training approach on skill retention and practice, direct client-level health outcomes and cost-effectiveness.

## Supplementary Material

ihag039_Supplemental_File

## Data Availability

All de-identified quantitative data, OSCE checklists/rubrics and analysis codes are available from the corresponding author. Redacted qualitative materials were available under controlled access to protect the participants’ confidentiality and privacy. Requests for data access can be made to the corresponding author with evidence of ethical approval and a data use agreement.
