# Supplementary material for: Piloting of a modular competency-based training to improve maternal and newborn health in Nepal: a mixed-methods study
Source: Int Health. 2026 Apr 23;18(6):920–8. doi: 10.1093/inthealth/ihag039 (PMC13728589; doi:10.1093/inthealth/ihag039)
Supplement: Supplementary file 1 [file ihag039_supplemental_file.docx]

**Supporting information**

## **S1 Table.** Summary of SHP/SBA modular LRP modules

| Module | Title | Rationale / Target Audience | Key Content Areas | Primary Methodology | Primary Assessment Methods |
| --- | --- | --- | --- | --- | --- |
| 1 | RMNC & Core Skills | Instil foundational principles of respectful, woman-centred care. This module is mandatory for all MNH providers to address a key gap in prior training. | RMNC, communication, Infection Prevention, pre-conception/antenatal and postnatal care, Postpartum family planning, Quality Improvement, health worker introduction, informed procedures, rights of mothers. | Interactive sessions, group discussions, and case studies. | Pre-test (True/False), post-test (MCQ). |
| 2 | Emergency Obstetric and Newborn First Aid | For all relevant frontline MNH service providers (Auxiliary Health Worker, Health Assistants, ANMs, Staff Nurses+) who manage emergencies. | Managing maternal/newborn emergencies, referral, and preparedness. | Skill stations, group-based learning, case studies, skill demonstrations, clinical simulation, and table-top exercises. | Knowledge pre-post questionnaires, OSCE pre-post. |
| 3 | Physiology and Management of Labour and Birth | Deepens understanding of normal Labour and birth, including monitoring, and recognizing deviations from the norm. | Normal Labour/birth physiology, Labour Care Guide use, core delivery skills, and in-utero physiological referral. | Interactive presentations, group discussions, and case studies. | True/False (learning needs), MCQ (certification), OSCE. |
| 4 | Complication Identification, Management, and Referral | Equips providers to identify, manage, and refer complications according to their scope of practice. | Gestational complications, IV cannulation, sick newborn referral, urine/acetic acid tests, sterile speculum examination, and perinatal mental health. | Pre- and post-assessment, simulation lab, competency-based training. | OSCE, case studies, and practical assessments. |
| 5 | Advanced MNH Providers | Specifically designed for physicians to address the need for cadre-specific training beyond the generic model. | Advanced management (Post Partum Haemorrhage bundle or drills, WHO safety checklist, post-operative complications, fistula prevention). | Blended learning, skills training, simulation. | Knowledge assessment, OSCE, simulation. |

**S2 Figure.** Conceptual Framework/Pathway to improve MNH in Nepal

**Abbreviations:** LRP (Learning Resource Package), MNH (Maternal and Newborn Health), OSCEs (Objective Structured Clinical Examinations), RMNC (Respectful Maternity and Newborn Care), SBA (Skilled Birth Attendant), SHP (Skilled Health Personnel), SDGs (Sustainable Development Goals), TWG (Technical Working Group).

1. **CONTEXT AND PROBLEM**

**Persistent Quality of Care Gaps in MNH in Nepal**

- Stagnating newborn mortality and high maternal mortality despite high facility births.
- Increased proportion of maternal deaths occurring within health facilities.
- One of the factors is sub-optimal competency of Skilled Birth Attendants (SBAs)

2. **INTEVENTION DEVELOPMENT AND IMPLEMENTATION (MIXED-METHODS STUDY)**

**PHASE 1: ASSESSMENT**

**Methods:** Stakeholder consultation, expert review and curriculum analysis

**Gaps:** Generic approach, overloaded curriculum, poor focus on RMNC, insufficient skills practice, weak post training support

**PHASE 2: DESIGN**

**Process:** Participatory approach involving TWG, iterative drafting, national consensus developed

**Resulting Intervention:** Competency based modular SHP/SBA LRP

**PHASE 3: PILOTING**

**Methods:** Purposive sampling, pre/post-test, OSCEs

**Immediate results:** Feasible, acceptable and scalable

3. **OUTPUTS (Immediate Results)**

- Developed SHP/SBA LRP: Complete, evidence-based training curriculum developed and pilot tested.
- Strengthened human resources: Cohort of MNH providers trained, proved feasibility and acceptability of SHP/SBA LRP.
- Established support system: Framework for post-training mentoring and coaching developed

**PROVIDER LEVEL**

**Knowledge:** Increased scores on MNH topics

**Skills:** Ability in managing emergencies (OSCEs)

**Confidence:** Enhanced self-efficacy

4. **OUTCOMES (Short to medium)**

**SERVICE DELIVERY AND SYSTEM**

**Practice:** Better adherence to clinical standards

**Respectful care:** Increased practice of RMNC

**System responsiveness:** Enhanced capacity to manage or refer

5. **LONG TERM IMPACTS (Improved MNH outcomes in Nepal)**

- Reduction in preventable maternal and newborn mortality and morbidity
- Strengthened MNH workforce and health system resilience
- Contribution to achieving national health goals and SDGs

**S3 File.** The STROBE and COREQ checklists were used in this study. (Completed reporting checklists for the observational [STROBE] and qualitative [COREQ] components of the study are provided)

**1. STROBE Statement—Checklist of items that should be included in reports of observational studies**

*Applicable to Phase III (The single-group pre-post pilot study).*

| Section/Topic | Item No. | Recommendation | Reported in Manuscript |
| --- | --- | --- | --- |
| Title and Abstract | 1 | (a) Indicate the study's design with a commonly used term in the title or the abstract. | Title: "Mixed-Methods Study"; Methods: "Single-group pre-post design". |
|  |  | (b) Provide in the abstract with an informative and balanced summary of what was done and what was found. | Abstract: Background, Methods, Results, and Conclusions are clearly summarised. |
| Introduction |  |  |  |
| Background/rationale | 2 | Explain the scientific background and rationale for the investigation being reported. | Introduction: "Stagnation paradox" in Nepal, gaps in SBA training, need for competency-based education. |
| Objectives | 3 | State specific objectives, including any prespecified hypotheses. | Introduction: Three specific objectives: develop LRP, pilot for feasibility/acceptability, estimate effects on knowledge/skills. |
| Methods |  |  |  |
| Study design | 4 | Present key elements of study design early in the paper. | Methods: "Three-phase design... Phase III involved pilot testing... single-group pre-post design". |
| Setting | 5 | Describe the setting, locations, and relevant dates, including periods of recruitment, exposure, follow-up, and data collection. | Methods: Gandaki Province (Matrisishu and Lamjung Hospital); June to July 2025. |
| Participants | 6 | (a) Give the eligibility criteria, and the sources and methods of selection of participants. | Methods: "24 participants were purposively selected to represent a mix of cadres... and facility types". |
| Variables | 7 | Clearly define all outcomes, exposures, predictors, potential confounders, and effect modifiers. | Methods: Outcomes: Knowledge (MCQ scores), Skills (OSCE scores), Feasibility, Acceptability. |
| Data sources/ measurement | 8 | For each variable of interest, give sources of data and details of methods of assessment (measurement). | Methods: Validated multiple-choice test (S4 File), OSCE stations with standardized rubrics, debriefing sessions. |
| Bias | 9 | Describe any efforts to address potential sources of bias. | Methods: "Standardized rubrics" used to ensure consistency; observer bias acknowledged in limitations. |
| Study size | 10 | Explain how the study size arrived at. | Methods: Pilot study with n=24; chosen for feasibility before larger trial. |
| Quantitative variables | 11 | Explain how quantitative variables were handled in the analyses. | Methods: Pre- and post-test scores compared; mean scores calculated. |
| Statistical methods | 12 | (a) Describe all statistical methods, including those used to control confounding. | Methods: Wilcoxon signed-rank tests were used for pre–post comparisons using Stata/SE 19. |
| Results |  |  |  |
| Participants | 13 | (a) Report numbers of individuals at each stage of study. | Results: "All the 24 participants completed the pilot training (100% completion rate)". |
|  |  | (b) Give reasons for non-participation at each stage. | Results: None; 100% adherence reported. |
| Descriptive data | 14 | (a) Give characteristics of study participants (eg demographic, clinical, social). | Results (Table 2): Ethnicity, qualification, designation, district, facility type. |
| Outcome data | 15 | Report numbers of outcome events or summary measures. | Results: Knowledge scores (median improvement 24.0%), Skills scores (Table 3). |
| Main results | 16 | (a) Give unadjusted estimates and, if applicable, confounder-adjusted estimates and their precision (eg, 95% confidence interval). | Results: Wilcoxon signed-rank test z-statistics, p-values (<0.001), and 95% CIs reported in Table 3. |
| Other analyses | 17 | Report other analyses done eg analyses of subgroups and interactions, and sensitivity analyses. | Results: Station-specific analysis (e.g., resuscitation 100%, PPH 95.8%). |
| Discussion |  |  |  |
| Key results | 18 | Summarise key results with reference to study objectives. | Discussion: "Study demonstrated that a modular... training programme can narrow the gap... feasible and acceptable". |
| Limitations | 19 | Discuss limitations of the study, considering sources of potential bias or imprecision. | Discussion: Small sample size (n=24), non-random, single province, potential observer bias, lack of control group. |
| Interpretation | 20 | Give a cautious overall interpretation of results considering objectives, limitations, multiplicity of analyses, results from similar studies, and other relevant evidence. | Discussion: Results align with global evidence; emphasizes need for supportive systems/mentorship for sustainability. |
| Generalisability | 21 | Discuss the generalisability (external validity) of the study results. | Discussion: Results suggest potential for national scale-up; adaptable to other LMICs. |
| Other information |  |  |  |
| Funding | 22 | Give the source of funding and the role of the funders for the present study. | Funding: RSTMH [2024 Grant], Health Training Centre, UNICEF, One Heart Worldwide. |

**2. COREQ (Consolidated Criteria for Reporting Qualitative Research) Checklist**

*Applicable to Phase I (Stakeholder consultations) and Phase III (Debriefing/Feedback)*

| Domain | Item No. | Guide Questions/Description | Reported in Manuscript |
| --- | --- | --- | --- |
| Domain 1: Research team and reflexivity |  |  |  |
| Personal Characteristics | 1 | Interviewer/facilitator: Which author/s conducted the interview or focus group? | Methods (Phase I & III): The research team (KBK & YA) conducted consultations and debriefings. |
|  | 2 | Credentials: What were the researcher's credentials? | Title Page & Funding: First author is a PhD student; co-authors include senior experts and trainers. |
|  | 3 | Occupation: What was their occupation at the time of the study? | Title Page: Researchers are affiliated with the Centre for International Health (LMU), NHTC, and Provincial Health Training Centre. |
|  | 4 | Gender: Was the researcher male or female? | Title Page: Mixed-gender team (e.g., Khim [Male], Yeshoda [Female]). |
|  | 5 | Experience and training: What experience or training did the researcher have? | Methods: Facilitators were "expert trainers" and TWG included "senior experts". |
| Relationship with participants | 6 | Relationship established: Was a relationship established prior to study commencement? | Methods: Participants were recruited from specific facilities; professional relationships existed given the authors' roles in NHTC/Directorate, though not explicitly detailed as "prior relationships." |
|  | 7 | Participant knowledge of the interviewer: What did the participants know about the researcher? | Methods: Participants provided informed written consent, implying knowledge of the study purpose. |
|  | 8 | Interviewer characteristics: What characteristics were reported about the interviewer/facilitator? | Methods: Described as "expert trainers" and TWG members. |
| Domain 2: Study design |  |  |  |
| Theoretical framework | 9 | Methodological orientation: What methodological orientation was stated to underpin the study? | Methods: "Inductive thematic analysis" was used for coding transcripts. |
| Participant selection | 10 | Sampling: How were participants selected? | Methods: "Purposive sampling" was used to represent a mix of cadres and facility types. |
|  | 11 | Method of approach: How were participants approached? | Methods: Through the NHTC/Provincial Health Directorate channels; invited to participate in workshops/training. |
|  | 12 | Sample size: How many participants were in the study? | Methods: Phase I: twelve stakeholders; Phase III: twenty-four participants. |
|  | 13 | Non-participation: How many people refused to participate or dropped out? | Results: 100% completion rate (n=24) for the pilot. |
| Setting | 14 | Setting of data collection: Where was the data collected? | Methods: Two sites in Gandaki Province (Matrisishu and Lamjung Hospital). |
|  | 15 | Presence of non-participants: Was anyone else present? | Not applicable/Not stated: Only researchers and participants during debriefings. |
|  | 16 | Description of sample: What are the important characteristics of the sample? | Results (Table 2): Detailed breakdown of ethnicity, qualification, designation, district, and facility type. |
| Data collection | 17 | Interview guide: Were questions, prompts, guides provided by the authors? | Methods: "Structured workshops," "in-depth consultations," and "structured debriefing sessions" were used. |
|  | 18 | Repeat interviews: Were repeat interviews carried out? | Methods: Iterative process with TWG meetings (6 meetings) and multiple workshops. |
|  | 19 | Audio/visual recording: Did the research use audio or visual recording? | Methods: Sessions were "audio recorded". |
|  | 20 | Field notes: Were field notes made during and/or after the interview or focus group? | Methods: Feedback was "recorded and incorporated". |
|  | 21 | Duration: What was the duration of the interviews or focus group? | Methods: Training was 36 days; specific duration of interview sessions is not explicitly stated but part of the training schedule. |
|  | 22 | Data saturation: Was data saturation discussed? | Methods: Phase II process continued until "consensus was reached". |
|  | 23 | Transcripts returned: Were transcripts returned to participants for comment? | Not explicitly stated: However, the participatory validation process (Phase II) implies feedback loops. |
| Domain 3: Analysis and findings |  |  |  |
| Data analysis | 24 | Number of data coders: How many data coders coded the data? | Methods: "Two analysts independently coded the transcripts". |
|  | 25 | Description of the coding tree: Did authors provide a description of the coding tree? | Methods: Described as "Inductive thematic analysis" resolving differences by consensus. |
|  | 26 | Derivation of themes: Were themes identified in advance or derived from the data? | Methods: Themes were derived from the data (inductive). |
|  | 27 | Software: What software, if applicable, was used to manage the data? | Not explicitly stated: Manual coding or standard office tools implied; Statistical software Stata 19 used for quantitative. |
|  | 28 | Participant checking: Did participants provide feedback on the findings? | Methods: "Participatory validation process" ensured alignment with national priorities. |
| Reporting | 29 | Quotations presented: Were participant quotations presented to illustrate the themes? | Results: Yes, e.g., "confidence moved from [her] head to [her] hands," "care for the mother, not just deliver the baby". |
|  | 30 | Data and findings consistent: Was there consistency between the data presented and the findings? | Results: Qualitative findings (barriers/enablers) align with the discussion on mentorship and resources. |
|  | 31 | Clarity of major themes: Were major themes clearly presented in the findings? | Results: Themes clearly categorized: "Feasibility and Acceptability," "Relevance to Practice," "Impact on RMNC," "Barriers". |
|  | 32 | Clarity of minor themes: Is there a description of diverse cases or minor themes? | Results: Discussion of "senior staff assuming leadership" vs "teamwork" highlights nuance in group dynamics. |

**S4 File.** A questionnaire was used for the pre- and post-training assessments. (Contains the set of multiple-choice questions administered to participants before and after the training for module one and skill checklist for module 2)

# **Module 1: Pre-course/ midcourse questionnaire**

Instructions: Write the letter of the single best answer to each question in the blank next to the corresponding number on the attached answer sheet.

1. The life cycle of continuum of care includes:

a. Pregnancy, ANC, Labour and delivery, PNC, Family planning, immunization, and nutrition.
b. Preconception, Pregnancy, ANC, Labour and delivery, PNC, Family planning, immunization, and nutrition.
c. Preconception, Pregnancy, ANC, Labour and delivery, PNC, Family planning
d. Pregnancy, ANC, labour and delivery, Postnatal period

2. Folic acid supplementation is essential for:

a. Helping the body to manufacture Iron
b. Protects the body from infection.
c. Prevents Anaemia.
d. Prevent Neural tube defects.

3. The dosage of Folic acid provided during Preconception is:

a. 400 mcg for 3 months before conception
b. 200 mcg for 3 months before conception
c. 800 mcg for 3 months before conception
d. 400 mcg for 3 months after conception

4. The estimated date of Delivery (EDD) is calculated:

a. from the first day of the last menstrual period
b. from the last day of the last menstrual period
c. from the middle of the month of the last menstrual period
d. from the beginning of the month of the last normal menstrual period

5. The fundus usually reaches the umbilicus at:

a. 22 weeks
b. 22 to 24 weeks
c. 16 weeks
d. none of the above

6. The woman’s blood pressure should be taken:

a. at the first antenatal visit
b. at the second antenatal visit
c. at each antenatal visit
d. only if she has protein in her urine.

7. Fetal well-being is assessed:

a. by listening to the foetal heart rate
b. foetal movements felt during abdominal palpation
c. by comparing uterine size to the gestational age of the foetus
d. all the above

8. The lie of the foetus should be checked:

a. in the first trimester of pregnancy
b. in the second trimester of pregnancy
c. in the third trimester of pregnancy
d. all the above

9. The woman’s haemoglobin should be checked:

a. at every antenatal visit
b. at the first and last visit
c. at the first visit and, if normal, again at 28 weeks gestation
d. only if the woman has clinical signs of anaemia.

10. Urine should be checked for protein:

a. at every antenatal visit
b. at the first antenatal visit
c. at the last antenatal visit
d. only if the woman’s blood pressure is elevated.

11. A birth plan should be developed:

a. only if the woman says she wants one.
b. only for women who are likely to experience a complication.
c. in consultation with each antenatal client at the first antenatal visit
d. in consultation with each antenatal client at the last antenatal visit
12. Dizziness or fainting during pregnancy, accompanied by fatigue, pallor, breathlessness, and rapid heartbeat may indicate:
a. ruptured ectopic pregnancy.
b. food poisoning
c. severe anaemia
d. heart disease

13. An important component of antenatal care for the HIV positive woman involves:

a. counselling her about the breastfeeding and replacement feeding so that she can make an informed choice.
b. telling her that she must not breastfeed.
c. telling her that she must not to bottle feed.
d. advising her to use a commercial formula

14. Schedule of 8 ANC visits are as follow:

a. upto 12 wks, 16 wks, 24 wks, 28 wks, 32 wks, 34 wks, 36 wks, 40 wks
b. upto 12 wks, 16 wks, 20-24 wks, 22 wks, 32 wks, 30-34 wks, 36 wks, 40 wks
c. upto 12 wks, 16 wks, 20-24 wks, 28 wks, 32 wks, 34 wks, 36 wks, 38-40 wks
d. upto 12 wks, 16 wks, 20-24 wks, 22 wks, 32 wks, 30-34 wks, 36 wks, 40 wks

15. The postpartum woman should be:

a. asked which family planning methods she has used and whether she wants to use a method in the future.
b. told that family planning is not necessary during the immediate postpartum period.
c. told that she must begin using a family planning method immediately if she is not fully breastfeeding.
d. all the above

16. Following the birth, the fundus:

a. decreases about 3 cm/day for the first 9-10 days.
b. decreases about 2 cm/day for the first 9-10 days.
c. decreases about 1 cm/day for the first 9-10 days.
d. increases first then decreases.

17. Advise and counseling should be provided to all postpartum women about:

a. hygiene
b. hygiene and nutrition
c. hygiene, nutrition, rest, and sleep
d. hygiene, nutrition, rest and sleep, family planning, and breastfeeding

18. Women who rely on lactational amenorrhea as a method of family planning should be advised that:

a. there is little risk of becoming pregnant for as long as breast is continued
b. there is no risk of becoming pregnant for as long as breastfeeding is continued
c. there is no risk of becoming pregnant if the baby is fed drinks/food other than breastmilk.
d. there is a risk of ovulating and becoming pregnant if the baby is fed drinks/food other than breastmilk.

19. For the prevention of anaemia in the postpartum period:

a. iron 60 mg + folate 400 mcg should be taken by mouth once daily for one month.
b. iron 60 mg + folate 400 mcg should be taken by mouth once daily for two months.
c. iron 60 mg + folate 400 mcg should be taken by mouth once daily for three months.
d. iron 60 mg should be taken by mouth once daily for three months.

20. The scheduled PNC visits are as follows:

a. 24 hrs, 3 days, 7 days, 42 days
b. upto 24 hrs, 3 days, 7-14 days, 42 days
c. within 24 hrs, 3 days, 7 days, 14 days
d. at 24 hrs, 3 days, 7-14 days, 42 days

21.The easiest place to break the disease transmission cycle is:

a. Reservoir
b. Mode of transmission
c. Place of entry
d. Susceptible host
22. Sterilized equipment can be used for ………. Days, if storage properly.
a. 7 days
b. 10 days
c. 14 days
d. 21 days
23. Respectful maternity and newborn care include:
a. Non consented care
b. Discrimination based on caste
c. Freedom from harm
d. Non confidential care
24. SPIKES model for breaking bad news in order includes:
a. Summary, Perception, Invitation, Empathy and Setting
b. Setting. Position, Invitation, Knowledge, and Summary
c. Setting, Perception, Invitation, Knowledge, Emotion and Summary
d. Setting, Perception, Invitation, Knowledge, Empathy and Summary
25. Family planning education and counselling should be provided during
a. Antenatal, intranatal, and postnatal period
b. Intranatal and postnatal period
c. Only in postnatal period
d. Only in postnatal period

**S5 File: Standardised Objective Structured Clinical Examination (OSCE) Assessment Rubrics (Modules 1–3)**

**1. Overview of the Assessment Framework**

The Objective Structured Clinical Examinations (OSCEs) used in this pilot implementation were developed *de novo* by the Technical Working Group (TWG). The stations evaluate the "Shows How" tier of Miller’s Pyramid of Clinical Competence. The checklists below represent the core standardised rubrics used across the modular Learning Resource Package (LRP) for Modules 1, 2, and 3, encompassing foundational assessments, emergency obstetric first aid, and physiological birth care.

Standardised Binary Scoring Logic:

- Performed Correctly (1 Point): The clinical step was executed accurately, in the correct sequence, and aligned with standard clinical protocols.
- Not Performed / Incorrect (0 Points): The clinical step was omitted entirely, performed out of sequence, or executed incorrectly.

**2. Core Standardised Rubrics by Competency Domain**

**Module 1: Foundational Clinical Assessment & Respectful Care**

**Station 1: Antenatal Abdominal Examination *Focus: Preparation, inspection, and systematic palpation.***

| Step | Performance Indicator (Criteria) | Score (0 or 1) |
| --- | --- | --- |
| 1.1 | Asks the mother to empty her bladder prior to examination. |  |
| 1.2 | Helps the mother onto the examination table, asks to uncover her abdomen, and positions her on her back. |  |
| 1.3 | Inspects the abdomen observes shape and size, notes abdominal scars, linea nigra, and striae gravidarum. |  |
| 1.4 | Measures fundal height accurately with a measuring tape. |  |
| 1.5 | Conducts fundal palpation applies gentle but firm pressure to assess the consistency and mobility of the fetal part (differentiating buttocks from head). |  |
| Total | Station Mastery Score (Max 5) | /5 |

**Station 2: Respectful Maternal and Newborn Care (RMNC) *Focus: Interpersonal communication, patient rights, and ethical clinical practice.***

| Step | Performance Indicator (Criteria) | Score (0 or 1) |
| --- | --- | --- |
| 2.1 | Ensures the client's right to freedom from harm and ill-treatment. |  |
| 2.2 | Ensures the right to information, informed consent, and respect for choices and preferences (including allowing a companion of choice during maternity care and refusal of medical procedures). |  |
| 2.3 | Maintains the right to privacy and confidentiality throughout the examination. |  |
| 2.4 | Treats the client (and newborn) as their own person from the moment of birth, with dignity and respect. |  |
| 2.5 | Delivers equitable care, ensuring the right to equality and freedom from discrimination. |  |
| 2.6 | Advocates for the client's right to healthcare and the highest attainable level of health. |  |
| Total | RMNC Integrated Score (Max 6) | /6 |

**Module 2: Emergency Obstetric and Newborn First Aid**

**Station 1: Airway and Breathing Management *Focus: Immediate resuscitation and respiratory stabilisation.***

| Step | Performance Indicator (Criteria) | Score (0 or 1) |
| --- | --- | --- |
| 1.1 | Clears the airway of any visible obstructions. |  |
| 1.2 | Maintains correct position utilising chin-lift or jaw-thrust manoeuvres. |  |
| 1.3 | Determines proper size and measurement before adjuncts are applied (oropharyngeal or nasopharyngeal airway). |  |
| 1.4 | Initiates high-flow oxygen support (4–6 litres per minute). |  |
| 1.5 | If not breathing spontaneously, initiates and maintains proper bag and mask ventilation. |  |
| Total | Station Mastery Score (Max 5) | /5 |

**Station 2: Shock Management *Focus: Rapid identification and stabilisation of hypovolaemic or septic shock.***

| Step | Performance Indicator (Criteria) | Score (0 or 1) |
| --- | --- | --- |
| 2.1 | Assesses airway patency and administers oxygen (6–8 litres/min). |  |
| 2.2 | Assesses uterine tone and initiates uterine massage if indicated. |  |
| 2.3 | Initiates IV fluid replacement (Isotonic solution + Oxytocin). |  |
| 2.4 | Performs urinary catheterisation to monitor output. |  |
| 2.5 | Monitors vital signs continuously and evaluates response to resuscitation. |  |
| Total | Station Mastery Score (Max 5) | /5 |

**Station 3: Bimanual Compression *Focus: Internal uterine compression to manage severe postpartum haemorrhage.***

| Step | Performance Indicator (Criteria) | Score (0 or 1) |
| --- | --- | --- |
| 3.1 | Performs hand hygiene and dons’ sterile surgical gloves. |  |
| 3.2 | Ensures RMNC, providing privacy and explaining the procedure to obtain consent. |  |
| 3.3 | Places a closed fist in the anterior vaginal fornix. |  |
| 3.4 | Uses the abdominal hand to compress the posterior uterine wall against the internal fist. |  |
| 3.5 | Maintains continuous pressure until bleeding is fully controlled. |  |
| Total | Station Mastery Score (Max 5) | /5 |

**Station 4: Aortic Compression *Focus: Emergency temporising measure for life-threatening pelvic bleeding.***

| Step | Performance Indicator (Criteria) | Score (0 or 1) |
| --- | --- | --- |
| 4.1 | Applies firm, downward pressure via a closed fist just above the umbilicus (slightly to the left). |  |
| 4.2 | Simultaneously palpates the femoral pulse to confirm vascular occlusion. |  |
| 4.3 | Verifies that the femoral pulse is non-palpable during compression. |  |
| Total | Station Mastery Score (Max 3) | /3 |

**Station 5: Condom Tamponade (UBT) *Focus: Uterine Balloon Tamponade application for refractory haemorrhage.***

| Step | Performance Indicator (Criteria) | Score (0 or 1) |
| --- | --- | --- |
| 5.1 | Aseptically assembles the condom over the catheter and secures it tightly without strangulating the inflow. |  |
| 5.2 | Inserts the catheter high into the uterine cavity. |  |
| 5.3 | Inflates the condom with 300–500 ml of sterile saline. |  |
| 5.4 | Maintains oxytocin infusion and monitors vital signs and bleeding every 15–30 minutes. |  |
| 5.5 | Follows the correct deflation protocol (gradual removal after 12–24 hours). |  |
| Total | Station Mastery Score (Max 5) | /5 |

**Station 6: Non-pneumatic Anti-Shock Garment (NASG) Application *Focus: Lower-body counter-pressure to reverse hypovolaemic shock.***

| Step | Performance Indicator (Criteria) | Score (0 or 1) |
| --- | --- | --- |
| 6.1 | Closes segment 1 (ankles) through 6 (abdomen) sequentially upwards. |  |
| 6.2 | Performs the "snap-test" to ensure adequate tightness of the garment. |  |
| 6.3 | Ensures haemodynamic stability for at least 2 hours before initiating removal. |  |
| 6.4 | Removes garment sequentially starting from segment 1, checking vital signs between opening each segment. |  |
| Total | Station Mastery Score (Max 4) | /4 |

**Station 7: Severe Pre-eclampsia / Eclampsia *Focus: Seizure management and maternal stabilisation.***

| Step | Performance Indicator (Criteria) | Score (0 or 1) |
| --- | --- | --- |
| 7.1 | Protects airway (left lateral position), provides oxygen, and prevents injury during active convulsions. |  |
| 7.2 | Administers Magnesium Sulphate loading dose (4g IV + 10g IM) with lidocaine admixture. |  |
| 7.3 | Administers Magnesium Sulphate maintenance dose (5g IM every 4 hours). |  |
| 7.4 | Conducts hourly assessments of respiratory rate (≥16), patellar reflexes, and urine output (≥30 ml/hr). |  |
| 7.5 | Prepares the antidote (Calcium Gluconate) at the bedside prior to administration. |  |
| Total | Station Mastery Score (Max 5) | /5 |

**Station 8: Kangaroo Mother Care (KMC) *Focus: Low-birthweight/preterm newborn thermal protection.***

| Step | Performance Indicator (Criteria) | Score (0 or 1) |
| --- | --- | --- |
| 8.1 | Counsels the mother on KMC benefits and ensures proper hand washing. |  |
| 8.2 | Ensures proper, open-front clothing for the mother and a cap/diaper for the baby. |  |
| 8.3 | Positions the baby skin-to-skin in a "frog-like" position with the head extended to keep the airway patent. |  |
| 8.4 | Wraps the baby securely to the mother's chest to prevent slipping. |  |
| 8.5 | Instructs the mother on appropriate feeding and sleeping positions while in KMC. |  |
| Total | Station Mastery Score (Max 5) | /5 |

**Station 9: Newborn Resuscitation *Focus: "The Golden Minute" for an asphyxiated newborn.***

| Step | Performance Indicator (Criteria) | Score (0 or 1) |
| --- | --- | --- |
| 9.1 | Dries and stimulates the newborn, clearing the airway (suctioning) if necessary. |  |
| 9.2 | Clamps the cord and assesses breathing and heart rate. |  |
| 9.3 | Applies the correct size bag and mask (size 0 or 1) achieving a proper seal. |  |
| 9.4 | Ventilates at a rate of 40 breaths per minute. |  |
| 9.5 | Reassesses heart rate and chest rise after 1 minute of positive pressure ventilation. |  |
| Total | Station Mastery Score (Max 5) | /5 |

**Station 10: Referral Management *Focus: Triage, communication, and safe transport.***

| Step | Performance Indicator (Criteria) | Score (0 or 1) |
| --- | --- | --- |
| 10.1 | Stabilises the patient haemodynamically prior to initiating transport. |  |
| 10.2 | Communicates effectively with the receiving facility to alert them of the incoming emergency. |  |
| 10.3 | Arranges appropriate transport and ensures a provider accompanies the patient if indicated. |  |
| 10.4 | Completes the referral slip comprehensively (demographics, vitals, treatments given, reason for referral). |  |
| Total | Station Mastery Score (Max 4) | /4 |

**Station 11: Puerperal Sepsis – Assessment and Management *Focus: Rapid systemic evaluation and identification of shock/sepsis.***

| Step | Performance Indicator (Criteria) | Score (0 or 1) |
| --- | --- | --- |
| 11.1 | Assesses airway (determines if patent or obstructed). |  |
| 11.2 | Assesses breathing (evaluates respiratory rate and pattern). |  |
| 11.3 | Assesses circulation (checks capillary refill time, pulse rate, blood pressure, and signs of refractory hypovolaemia). |  |
| 11.4 | Assesses disability (uses AVPU scale, checks for convulsions). |  |
| 11.5 | Evaluates the condition and exposes to explore (e.g., identifies continuous fever over 24 hours including the first day of delivery). |  |
| Total | Station Mastery Score (Max 5) | /5 |

**Module 3: Physiology and Management of Labour and Birth**

Station 1: Intrapartum Abdominal & Vaginal Examination *Focus: Advanced fetal assessment and labour monitoring.*

| Step | Performance Indicator (Criteria) | Score (0 or 1) |
| --- | --- | --- |
| 1.1 | Inspects the abdomen (observes shape, size, and previous surgical scars). |  |
| 1.2 | Measures fundal height in centimetres. |  |
| 1.3 | Accurately notes the time and duration of contractions (feeling the contraction when the mother acts for pain). |  |
| 1.4 | Performs Leopold's manoeuvres: Fundal palpation. |  |
| 1.5 | Performs Leopold's manoeuvres: Lateral palpation. |  |
| 1.6 | Performs Leopold's manoeuvres: Pelvic palpation. |  |
| 1.7 | Assesses for fetal head descent. |  |
| 1.8 | Auscultates fetal heart rate (FHR) and documents findings. |  |
| Total | Station Mastery Score (Max 8) | /8 |

**Station 2: Postpartum Haemorrhage (PPH) Bundle *Focus: Immediate clinical response to excessive postpartum bleeding****.*

| Step | Performance Indicator (Criteria) | Score (0 or 1) |
| --- | --- | --- |
| 2.1 | Executes early detection of postpartum haemorrhage. |  |
| 2.2 | Initiates immediate massage of the uterus. |  |
| 2.3 | Administers appropriate oxytocic drug (e.g., repeat 10 IU IM). |  |
| Total | Station Mastery Score (Max 3) | /3 |

**Station 3: Immediate Newborn Care / Skin-to-Skin *Focus: Thermal protection and maternal bonding.***

| Step | Performance Indicator (Criteria) | Score (0 or 1) |
| --- | --- | --- |
| 3.1 | Wraps the mother and newborn together securely using a long wrap. |  |
| 3.2 | Secures the wrap by making a knot in front of the mother's abdomen. |  |
| 3.3 | Instructs/assists the mother to put a loose blouse or dress over the baby to maintain thermal control. |  |
| Total | Station Mastery Score (Max 3) | /3 |
